# The levels of interleukin-6, serum calprotectin, and hypersensitive C-reactive protein in patients with Crohn's disease-related mucosal damage

**DOI:** 10.5937/jomb0-60136

**Published:** 2026-02-27

**Authors:** Chuanshuo Zhang, Fengning Zhou, Xiandong Cao, Chenyang Qiu, Mingdian Lu, Xin Yu, Pingping Xiang, Bo Yu, Bo Zhou

**Affiliations:** 1 The First Affiliated Hospital of Anhui Medical University, Department of General Surgery, Hefei City, Anhui Province, China; 2 Zhejiang Chinese Medical University, Digestive Diseases of Research Centre, Hangzhou, China; 3 Fudan University, Digestive Diseases Research Centre, Shanghai, China

**Keywords:** hypersensitive C-reactive protein, mucosal injury, interleukin-6, Crohn's disease, visokosenzitivni C-reaktivni protein, oštećenje sluzokože, interleukin-6, Kronova bolest

## Abstract

**Background:**

To evaluate the ability of calprotectin, hypersensitive C-reactive protein, and interleukin-6 to detect digestive tract mucosal injury in patients with Crohn's disease (CD).

**Methods:**

Fifty-two patients diagnosed with CD were selected. Faecal samples from the patients were collected to detect calprotectin (ELISA), and serum samples were collected to detect hs-CRP (immunoturbidimetry) and IL-6 (chemiluminescence). All patients with CD underwent colonoscopy or capsule endoscopy, according to the modified endoscopic severity index of CD (SES-CD). Comparisons of the differences in calprotectin, hs-CRP and IL-6 levels were made between the two groups, and receiver operating characteristic (ROC) curves were drawn to analyse the diagnostic efficacy (AUC) of each index and combined detection for mucosal injury.

**Results:**

In the Mucosal injury group, the calprotectin and hs-CRP levels were significantly greater than those in the Mucosal healing group. According to the ROC analysis, calprotectin had the highest AUC (0.93, 95% CI: 0.88-0.98), sensitivity (88.5%) and specificity (89.2%) when the critical value was greater than 250 mg/g. The AUC of the three indicators' combined detection (logistic regression model) rose to 0.96, which was noticeably superior to that of a single indicator (P&lt; 0.05).

**Conclusions:**

Interleukin-6, serum calprotectin, and hypersensitive C-reactive protein levels are significantly correlated with the degree of mucosal injury in patients with CD. Among them, calprotectin has the best diagnostic value. The combined detection of these three factors can significantly improve the recognition of mucosal injury in the active stage of CD.

## Abstract (sr)

**Uvod:**

Cilj je bio da se proceni sposobnost kalprotektina, visokosenzitivnog C-reaktivnog proteina i interleukina-6 da otkriju oštećenje sluzokože digestivnog trakta kod pacijenata sa Kronovom bolešću (KB).

**Metode:**

Izabrana su 52 pacijenta sa dijagnozom KB. Prikupljeni su uzorci stolice radi određivanja nivoa kalprotektina (ELISA metodom) i uzorci seruma radi merenja hs-CRP (imunoturbidimetrija) i IL-6 (hemiluminiscencija). Svi pacijenti su podvrgnuti kolonoskopiji ili kapsularnoj endoskopiji. Ocenjena je težina bolesti prema modifikovanom endoskopskom indeksu aktivnosti KB (SES-CD). Upoređivane su razlike u nivoima kalprotektina, hs-CRP i IL-6 između dve grupe, a zatim su nacrtane ROC krive radi analize dijagnostičke efikasnosti (AUC) svakog parametra i njihove kombinacije u otkrivanju oštećenja sluzokože.

**Rezultati:**

U grupi sa oštećenjem sluzokože, nivoi kalprotektina i hs-CRP su bili značajno viši nego u grupi sa izlečenom sluzokožom. Prema ROC analizi, kalprotektin je imao najveći AUC (0,93, 95% CI: 0,88-0,98), senzitivnost (88,5%) i specifičnost (89,2%) kada je granična vrednost bila veća od 250 mg/g. Kombinovana detekcija svih triju markera (logistički regresioni model) dala je AUC od 0,96, što je statistički značajno bolje od pojedinačnih pokazatelja (P&lt; 0,05).

**Zaključak:**

Nivoi interleukina-6, serumskog kalprotektina i visokosenzitivnog C-reaktivnog proteina značajno su povezani sa stepenom oštećenja sluzokože kod pacijenata sa KB. Od svih, kalprotektin ima najbolju dijagnostičku vrednost. Kombinovano određivanje ova tri parametra značajno poboljšava detekciju oštećenja sluzokože u aktivnoj fazi bolesti.

## Introduction

Although progression is slow, the active stage and the remission stage alternate, and there is a life-long tendency toward recurrence [1]. Gastroscopy and colonoscopy, as well as pathological tissue biopsy, are essential methods for detecting inflammation of the digestive tract mucosa in patients with Crohn's disease [2][3][4]. However, owing to their invasive nature, these methods are not widely accepted by patients. Therefore, finding an acceptable and simple method is significant for both disease diagnosis and condition monitoring. Recent studies [5][6][7] have shown that calprotectin is a relatively ideal noninvasive biomarker. Moreover, because its detection is noninvasive and can be repeated, it is more easily accepted by patients than the traditional gold standard method.

Hypersensitive C-reactive protein (hs-CRP) is a classic inflammatory indicator and can be used to assess the condition of patients with Crohn's disease. Serum interleukin-6 (IL-6) is a pleiotropic inflammatory factor [8]. Therefore, IL-6, an inflammatory biomarker, has been increasingly widely used in clinical practice. In patients with Crohn's disease, if digestive tract mucosal injury occurs, it is usually accompanied by intensified mucosal inflammatory activity and increased infiltration of inflammatory cells [9]. If drug treatment is not carried out in time, over time, it will evolve into irreversible intestinal injury, requiring surgical intervention [10].

Most patients with undamaged mucosa are in the quiescent stage of the disease and have a good prognosis. After the condition assessment, only long-term medication is needed to maintain clinical remission.

## Materials and methods

Fifty-two patients, including 28 males and 24 females, who were admitted to our hospital from June 2024 to June 2025, were selected as research subjects.

Basic data collection and grouping included sex, age, Montreal classification, and the completion status of digestive tract endoscopy. The involved lesion sites are classified as L1 (involving only the ileum), L2 (involving only the colorectum), L3 (involving both the ileum and the colorectum), and L4 (involving the jejunum and the digestive tract above it). L4 can coexist with L1 to L3 or exist alone. Disease behaviour can be classified into B1 (nonstenotic or nonpenetrating), B2 (stenotic), and B3 (penetrating). All B1 to B3 lesions can be combined with perianal lesions simultaneously (p). Mucosal uninjury was defined as no manifestations, such as mucosal ulcers, erosions or congestion, observed under digestive tract endoscopy.

### Detection of serum IL-6

Five millilitres of peripheral blood were extracted from the patient within twenty-four hours of admission, and the serum was separated. Using a BD flow cytometer CANTO II and a Jiangxi Saiji Biology detection kit, the amount of IL-6 was measured via a cytometric bead array (CBA).

### Calprotectin detection

Faeces were collected within 72 hours of patient admission. A faecal calcium determination kit and supporting instrument (model FR-101) produced by Guangzhou Fengrun Biology Co., Ltd. were used to detect the calprotectin content in the faeces via immunofluorescence chromatography technology.

### hs-CRP detection

Each patient had three millilitres of peripheral blood extracted within twenty-four hours of admission, and the blood was anticoagulated with EDTA. The hs-CRP content in the patient's whole blood was detected by scattering turbidimetry via the hypersensitive C-reactive protein assay kit of Shenzhen Pumen Technology Co., Ltd. and the Pumen PA-990 Pro instrument.

### Statistical analysis

The data were plotted via GraphPad Prism 8.3.0. The data were processed via SPSS 24.0 and MedCalc 20.0 software. Correlation analysis of the quantitative data was conducted via Spearman's rank correlation analysis. Qualitative data are expressed in terms of frequency and percentage.

## Results

### General information about patients

All 52 patients included in the study completed a colonoscopy and did not use biological agents within 8 weeks. Among them, 21 patients (40.4%) were in the mucosal undamaged group, including 12 males and 9 females. One patient underwent duo-denoscopy examination. There were 31 patients (59.6%) in the mucosal injury group, including 16 males and 15 females. Among them, 3 patients underwent duodenoscopy, and 1 patient underwent double-balloon enteroscopy (Table 1).

**Table 1:** Clinical case data.

Indicators	Uninjured	Injured	*P*
Age (Year)	31.1±9.6	34.2±10.2	0.174
Female (%)	12 (58.2)	16 (52.7)	0.696
Diagnosis Age (%)			0.70
A_1_	0 (0)	0 (0)	
A_2_	16 (76.2)	25 (80.7)	
A_3_			
Lesion site/n (%)			0.215
L_1_	8 (38.1)	6 (19.4)	
L_2_	3 (14.3)	4 (12.9)	
L_3_	9 (42.8)	21(67.7)	
L_3_+L_4_	1 (4.8)	0 (0)	
Disease behavior/n (%)			0.020
B_1_	11 (52.4)	16 (51.6)	
B_2_	4 (19.0)	14 (45.2)	
B_3_	5 (223.8)	0 (0)	
B_2_ + B_3_	1 (4.9)	1 (3.3)	
Perianal lesion (p)/*n* (%)	16 (72.5)	18 (55.9)	0.229

### Patients with undamaged mucosa and those with damaged mucosa

**Figure 1:**
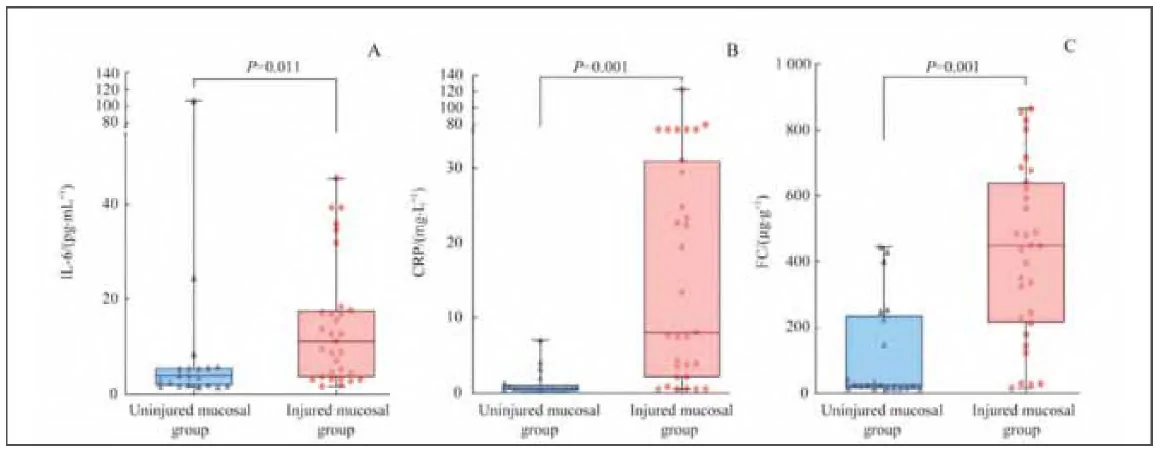
Interleukin-6, calprotectin, and hypersensitive C-reactive protein levels in the damaged mucosa group were compared. A. Interleukin-6; B. Hypersensitive C-reactive protein; C. Calprotectin.

Comparison and correlation analysis of the hypersensitive C-reactive protein, interleukin-6 and calprotectin levels revealed that in the group with mucosal injury, the levels of IL-6 [3.97 (1.93, 5.60) pg/L], hs-CRP [0.56 (0.51, 1.08) mg/L], and non-mucosal injury group calprotectin [25.80 (15.00, 235.45) μg/g] were noticeably greater than those in the other groups (all P<0.05, Figure 1). There were correlations between each pair of IL-6, hs-CRP and calprotectin levels (Table 2).

**Table 2:** Correlation investigation between calprotectin, hs-CRR and IL-6.

Indicators	Interleukin-6	Hypersensitive C-reactive protein	Calprotectin
Interleukin-6	-	0.726	0.388
Hypersensitive C-reactive protein	0.716	-	0.609
Calprotectin	0.388	0.609	-

### Analysis of factors influencing intestinal mucosal injury

The Hosmer Lemeshow test yielded P=0.954, indicating that this model was well fitted. Among IL-6, hs-CRP and calprotectin levels and disease behaviour, only the level of calprotectin was an independent influencing factor for whether the digestive tract mucosa was damaged in patients with Crohn's disease (P=0.012, Table 3).

**Table 3:** Multivariate logistic regression analysis of factors influencing gastrointestinal mucosal injury.

Indicators	Beta	Wald Statistic	Standard Error	P	95% Confidence Interval
Interleukin-6	-0.024	0.022	1.138	0.287	0.938 1.018
Hypersensitive C-reactive protein	0.324	0.213	2.48	0.116	0.924 2.108
Calprotectin	0.007	0.003	6.289	0.013	1.002 1.012
DB	0.172	0.593	0.084	0.774	0.373 3.788
Continuous variable	-2.325	1.362	2.916	0.089	

### Evaluation of indicators alone and in combination to assess mucosal damage in the digestive system

**Figure 2:**
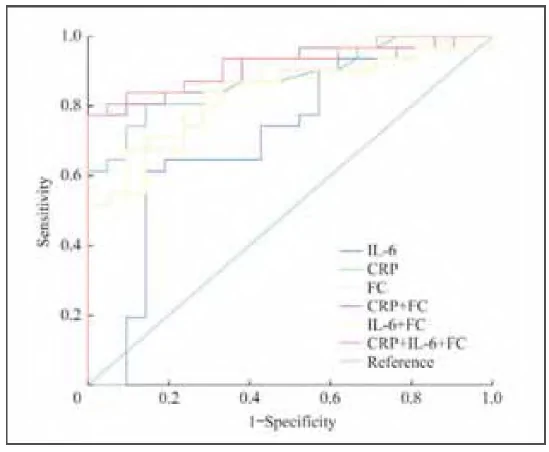
ROC curves of the joint detection of calprotectin, hs-CRP and IL-6 for evaluating gastrointestinal mucosal injury in CD patients.

The single indicators of interleukin-6, calprotectin, and hypersensitive C-reactive protein for assessing intestinal mucosal injury (all P<0.05) and the thresholds calculated on the basis of the Youden index were 7.59 pg/mg/L, 1.07 mg/L and 72.56 μg/g, respectively. When calprotectin + hs-CRP calprotectin + IL-6 and calprotectin + IL-6 + hs-CRP were combined, the AUC was 0.92 (Table 4 and Figure 2).

**Table 4:** Assessing gastrointestinal mucosal damage in CD patients using the individual and combination detection of IL-6, calprotectin, and hs-CRR

Item	Cut-off value	Sensitivity (%)	Specificity (%)	AUC	P	Youden index
Calprotectin + Hypersensitive<br>C-reactive protein	-	78	100	0.92 (0.84 0.99)	0.001	0.78
Calprotectin + Interleukin-6	-	69	92	0.85 (0.74 0.96)	0.001	0.59
cCalprotectin + Interleukin-<br>6 + Hypersensitive C-reactive<br>protein	-	78	100	0.93 (0.86 1.00)	0.001	0.78
Interleukin-6	7.59 pg**·**mL^-1^	62	87	0.72 (0.78 0.98)	0.008	0.48
Hypersensitive C-reactive<br>protein	1.07 mg**·**L^-1^	82	87	0.89 (0.89 1.00)	0.001	0.67
Calprotectin	72.56 mg**·**g^-1^	88	68	0.86 (0.67 0.93)	0.001	0.55

## Discussion

The prevalence of Crohn's disease has been increasing over the last 20 years, and the incidence of inflammatory bowel disease (IBD) has also been growing, increasing from 1.96 per 100,000 people to 3.14 per 100,000 people before reaching 44 per 100,000 people in 2024 [11]. More than 80% of patients develop the disease before the age of 40. When inflammation in the digestive tract of patients with this disease is out of control, irreversible intestinal damage often occurs, increasing surgical risk, complications and disability [12]. In addition to invasive examination methods such as gastroscopy, colonoscopy and tissue biopsy, current imaging techniques such as magnetic resonance enterography (MRE) of the small intestine also show good accuracy in the assessment of active diseases, but their detection costs are relatively high. It has increased the medical burden on patients [13]. Therefore, in this study, three noninvasive inflammatory markers were used to predict inflammation in the digestive tract mucosa by detecting changes in their levels.

Calprotectin is a calcium-zinc-binding protein that exists mainly in neutrophils. When inflammation occurs in the intestine, the increased permeability of the intestinal mucosa leads to the infiltration of neutrophils. It promotes their release of calprotectin, which can eventually be detected in faeces [14][15][16][17]. According to research reports on calprotectin in recent years, calprotectin has good clinical value in the diagnosis, activity assessment and therapeutic effect monitoring of inflammatory bowel disease. Moreover, it has many advantages, such as low cost, noninvasive detection, high acceptance by patients and convenient operation. Relevant studies have proposed that calprotectin can effectively reflect the degree of intestinal mucosal inflammation and can be used for inflammation assessment in patients [18][19][20]. When the calprotectin threshold was 82.55 μg/g, the predictive sensitivity and specificity for mucosal injury in patients with CD were 87% and 67%, respectively. However, owing to the lack of traceable quality control substances, standardised calibrators and corresponding quality control systems, the accuracy of calprotectin test results is difficult to guarantee. Moreover, the results of this indicator are affected by many factors, from pretreatment before analysis to actual detection, which limits the clinical value of this indicator.

hs-CRP is a classic indicator for evaluating inflammation in patients with Crohn's disease [21][22][23]. It is closely related to inflammatory factors and has a strong positive correlation with the activity of Crohn's disease [24]. This study also demonstrated correlations among the three inflammatory indicators. When the hs-CRP threshold was 2.06 mg/L, the predictive sensitivity and specificity for mucosal injury were 81% and 86%, respectively. Since hs-CRP is a nonspecific inflammatory marker and the reference range for the normal population is 0-8 mg/L, the clinical value of using a single indicator is relatively low [25][26][27].

In the pathogenesis of Crohn's disease, IL-6 can activate the nuclear factor B (NF-B) pathway, increase the permeability of epithelial cells, and cause intestinal mucosal microcirculation disorders. However, its value in identifying intestinal mucosal injury is relatively small [28][29][30]. This finding indicates that its increase has a specific value in judging the state of mucosal injury, but is smaller than that of hs-CRP and calprotectin.

When calprotectin + hs-CRP was detected together, the AUC of damaged digestive tract mucosa was 0.91, indicating relatively high diagnostic accuracy. When calprotectin + hs-CRP+IL-6 were combined for detection, the AUC for predicting damage to the digestive tract mucosa reached 0.92, which was greater than the results of each single and double combined detection. Moreover, the sensitivity and specificity of both methods were consistent, and the diagnostic efficacy was comparable.

## Conclusion

Calprotectin and hs-CRP can better determine mucosal damage to the digestive tract in patients with Crohn's disease, and they have the advantage of being noninvasive. While reducing the intestinal damage and economic burden of patients, it also improves the acceptance of examination by patients, which is conducive to timely monitoring of the course and therapeutic effect of CD and enhances the efficiency of follow-up. It is worthy of clinical application and promotion.

## Dodatak

### Conflict of interest statement

All the authors declare that they have no conflict of interest in this work.
